# Effectiveness and Safety of Hydroxyurea in the Treatment of Sickle Cell Anaemia Children in Jos, North Central Nigeria

**DOI:** 10.1093/tropej/fmz070

**Published:** 2019-10-14

**Authors:** Akinyemi O D Ofakunrin, Stephen Oguche, Kehinde Adekola, Edache S Okpe, Tolulope O Afolaranmi, Ijeoma N Diaku-Akinwumi, Ayuba I Zoakah, Atiene S Sagay

**Affiliations:** 1 Department of Paediatrics, College of Health Sciences, University of Jos/Jos University Teaching Hospital, Jos, Nigeria; 2 Division of Hematology-Oncology, Northwestern University, Feinberg School of Medicine, Chicago, IL, USA; 3 Department of Community Medicine, College of Health Sciences, University of Jos/Jos University Teaching Hospital, Jos, Nigeria; 4 Department of Paediatrics, Lagos State University Teaching Hospital, Lagos, Nigeria; 5 Department of Obstetrics and Gynaecology, College of Health Sciences, University of Jos/Jos University Teaching Hospital, Jos, Nigeria

**Keywords:** sickle cell anaemia, hydroxyurea, effectiveness, safety, adverse events, Nigeria

## Abstract

**Background:**

Hydroxyurea has been shown to positively modify sickle cell disease pathogenesis, but its use is low among Nigerian sickle cell anaemia (SCA) patients because of effectiveness and safety concerns.

**Methods:**

We conducted a quasi-experimental study to evaluate the effectiveness and safety of hydroxyurea in 54 SCA children aged 4–17 years. Clinical and haematological parameters were compared at baseline and 12 months after hydroxyurea therapy. The participants were monitored for adverse events. The parameters were compared using relative risk and Wilcoxon Signed-Rank Test.

**Results:**

The number of subjects who had more than two episodes of painful crises reduced from 27 (50%) to 2 (2.7%) (*p* < 0.001), while those who had acute chest syndrome reduced from 6 (11.1%) to 0 (0.0%; *p* < 0.001). The risk of being transfused more than once was 0.11 times the risk in the 12 months period preceding therapy (95% CI = 0.02–0.85; *p* = 0.016). Similarly, the risk of hospital stay >7 days was 0.08 times the risk at the baseline (95% CI = 0.02–0.24; *p* < 0.0001). The median haematocrit and percentage foetal haemoglobin increased from 26 to 28% and 7.8 to 14%, respectively (*p* < 0.0001). A dose-dependent but reversible leucopenia was observed among six children (11.1%), otherwise, hydroxyurea was safe in the study population.

**Conclusion:**

Hydroxyurea is effective and safe in SCA children in Jos, Nigeria. The findings could strengthen educational programme aimed at improving the utilization of hydroxyurea among SCA children.

## INTRODUCTION

Sickle cell disease (SCD) is a genetic blood disorder with increasing public health importance worldwide [1]. Globally, over 300 000 children are born annually with SCD, of which over 70% of the births occur in Sub-Saharan Africa. Majority of the affected children in Sub-Saharan Africa die before the age of 5 years from poor access to standard therapy [2–4]. Nigeria has the highest burden of SCD worldwide with about 150 000 affected newborns delivered annually [5–7].

Sickle cell anaemia (SCA), the homozygous state of SCD, is a chronic and progressively debilitating condition that is characterized by ongoing haemolytic anaemia, recurrent acute painful vaso-occlusive events and other complications [8, 9]. The complications include organs dysfunction which begins early in life and worsen over time [9]. SCA is associated with poor quality of life among those affected and the natural history usually ends in early mortality [9, 10]. Bone marrow transplant is a known cure for SCA but the procedure is hampered by restricted availability, high cost, difficulty in getting a suitable donor and associated life-threatening complications [11]. Hydroxyurea has, however, emerged as a major breakthrough in the therapy of SCD. It is the only currently available approved agent that is capable of modifying the disease pathogenesis and its use has transformed the treatment of SCD worldwide [12–14]. Clinical efficacy of hydroxyurea in the reduction of sickle cell crisis, stroke incidence, chronic organ damage and overall mortality has been reported [12–17]. Its safety in children as young as 6–9 months of age has also been shown by recent studies [16, 18]. A recent study also demonstrated the effectiveness of moderate fixed-dose hydroxyurea in primary stroke prevention while another reported a decrease in the incidence of malaria episodes following hydroxyurea therapy [19, 20].

However, in Nigeria, the use of hydroxyurea is low among SCA patients because of apprehension related to the effectiveness and safety of the medication [21]. This study evaluated the effectiveness and safety of hydroxyurea in children with SCA.

## METHODOLOGY

### Study location and design

This quasi-experimental study was conducted between March 2017 and May 2018 at the Paediatric Sickle Cell Clinic of the Jos University Teaching Hospital, a 525-bed public tertiary health facility in Jos, North Central Nigeria.

### Sampling technique

Following the recruitment of children with SCA who met the inclusion criteria, computer-generated table of random number was used to select the study subjects from the serialized list of the eligible patients.

### Inclusion criteria

SCA (Hb SS) children aged 4–17 years who were in steady state (no crisis, infection or fever for at least 4 weeks and no blood transfusion in the preceding 3 months) [22]; who have had two or more painful vaso-occlusive crises requiring parenteral analgesia within the previous 12 months; and were never previously on hydroxyurea were included.

### Exclusion criteria

Children with severe malnutrition, human immunodeficiency virus infection, malignancy or other chronic conditions that could potentiate hydroxyurea toxicity were excluded.

### Sample size determination

This was estimated using an appropriate sample size formula for an interventional study [23]. Based on the proportion of mean foetal haemoglobin from a previous study [24], standard normal deviate at 95% CI and the statistical power of 80%, a minimum sample size of 54 was obtained after an attrition rate of 10% was added.

### Data collection

#### Baseline

A semi-structured interviewer administered proforma was used to obtain the sociodemographic data from the participants. The socioeconomic status of the family was classified using the Olusanya *et al.* [25] classification which was based on the occupational and educational status of the parents.

The clinical histories of the patients over a 12-month period prior to the commencement of the study were extracted from the patients’ records. The clinical parameters obtained included the number of vaso-occlusive crises (pain in the extremities, back, abdomen, chest or head for which no other explanation could be found; that requires the use of parenteral analgesic), hospitalizations and the duration of hospitalization, acute chest syndrome (defined as a new infiltrate on the chest X-ray accompanied by respiratory symptoms and/or fever) and blood transfusions. General physical examination was carried out to detect any abnormality. At the commencement of the study, baseline haematology profile (haematocrit level, white blood cell count and platelet count) was measured for each of the subjects using an automated haematology analyser (Sysmex XN 550) while percentage foetal haemoglobin was assayed using High Performance Liquid Chromatography (VARIANT™ II, Bio-Rad Laboratories, Inc., CA). Information regarding the risks and benefits of hydroxyurea was provided to the older participants and the parents.

### Intervention phase

Hydroxyurea (Oxyurea) was commenced at 20 mg/kg and increased by 5 mg/kg after every 8 weeks to a maximum dose of 35 mg/kg (not exceeding 1000 mg) or 2.5–5.0 mg/kg below the dosage where haematologic toxicity was observed [24]. The hydroxyurea formulation used (Oxyurea) is available in 100, 250 and 500 mg capsules and were provided to the participants free of cost. When required, liquid formulations were prepared from the capsules by the hospital pharmacists. A 2-week supply was prepared each time and the beneficiaries were asked to store the liquid formulation inside +2 to +8°C refrigerator or at room temperature [26]. The study participants were treated with hydroxyurea and followed up for 12 months. They were clinically evaluated every month at a fixed regular interval or as the needs arise for the stated clinical parameters over a 12-month period. Other clinical events including known side effects of hydroxyurea were monitored with interval history and physical examination every month or as the needs arise. Full blood count (FBC) and percentage foetal haemoglobin were measured after 12 months of hydroxyurea therapy, in addition to 4 weekly serial FBC carried out to monitor participants for haematologic toxicity. Adherence to hydroxyurea therapy was reinforced by counselling of the older study participants and the parents. The participants were instructed to bring leftover pills or liquid formulations at each visit which were counted or measured to monitor adherence [27]. Adherence was said to be good if <10% of the prescribed dosage was returned. All the study participants had folic acid (5 mg/day), sulphadoxine/pyrimethamine (monthly) and were advised to take liberal oral fluids.

### Grading of responses

Effectiveness of hydroxyurea was determined using the changes in the clinical and haematological parameters observed between baseline and 12 months of hydroxyurea therapy.

The safety of hydroxyurea was assessed by monitoring haematologic toxicity and adverse clinical events during therapy. Haematologic toxicity was said to be present if the haematocrit level was <15% (severe anaemia), or a white blood cell count <4 × 10^9^/l (leucopenia), or a platelet count <80 × 10^9^/l (thrombocytopenia) [28].

### Ethical consideration

Ethical clearance was obtained from the Research and Ethics Committee of the Jos University Teaching Hospital. Written informed consents were obtained from the parents as well as assents from the study participants aged 7 years and above.

### Data analysis

The data obtained were processed and analysed using Stata 14 statistical software package. The quantitative variables such as age, weight, maximum dose of hydroxyurea, haematologic parameters were summarized using mean ± SD where assumptions of normality were fulfilled, otherwise median and interquartile range were used. The clinical parameters 12 months before and after hydroxyurea therapy were compared using relative risk. Wilcoxon Signed-Rank Test was used to compare the differences in the median values of the haematologic parameters at baseline and after 12 months of hydroxyurea therapy. A 95% CI was used and a *p*-value of < 0.05 was considered statistically significant.

## RESULTS

A total of 54 children with SCA (30 males and 24 females; age range: 4–17 years) participated in the study out of which none dropped out. The mean age of the study population was 8.47 ± 3.89 years. The mean maximum dosage of hydroxyurea was 29.7 ± 5.1 mg/kg and 51 (94.4%) of the subjects had good medication adherence (Table 1). The distribution of the phenotypic characteristics of the subjects is as shown in Table 2.

**Table 1:** Characteristics of the subjects (n = 54)

Parameters	Frequency (*n* = 54)	Percentage
Sex		
Male	30	55.6
Female	24	44.4
Age group (years)		
4–7	24	44.4
8–11	16	29.6
12–15	11	20.4
>15	3	5.6
Mean age ± SD^a^ (years)	8.47 ± 3.89	
Socioeconomic status of family		
Low	16	29.6
Middle	21	38.9
Upper	17	31.5
Mean baseline weight ± SD, kg	24.3 ± 10.7	
Mean maximum hydroxyurea dose ± SD, mg/kg	29.7 ± 5.1	
Adherence to hydroxyurea therapy		
Good	51	94.4
Poor	3	5.6

a Standard deviation.

**Table 2:** Distribution of the phenotypic characteristics of the subjects (n = 54)

Parameters	Pre-hydroxyurea	Post-hydroxyurea
	Frequency (percentage)	Frequency (percentage)
Number of VOC		
0–1	0 (0)	50 (92.6)
2–3	42 (77.8)	4 (7.4)
>3	12 (22.2)	0 (0)
Number of hospitalizations		
0–1	6 (11.1)	50 (92.6)
2–3	38 (70.3)	4 (7.4)
>3	10 (18.6)	0 (0)
Number of blood transfusion		
0	28 (51.8)	50 (92.6)
1	17 (31.5)	3 (5.5)
>1	9 (16.7)	1 (1.9)
Acute chest syndrome		
0	48 (88.9)	54 (100.0)
1	5 (9.2)	0 (0)
>1	1 (1.9)	0 (0)

### Clinical and haematological parameters of the subjects before and after hydroxyurea therapy

There was a significant reduction in the occurrence of vaso-occlusive crises, blood transfusions, hospitalizations, duration of hospitalization and episodes of acute chest syndrome from the baseline values compared with the values after 12 months of hydroxyurea (Table 3).

**Table 3:** Clinical and haematological parameters of the subjects before and after hydroxyurea therapy (n = 54)

Parameters	Pre-HUT	Post-HUT	RR	95% CI	*p*
	Frequency (%)	Frequency (%)			
Clinical					
Number of VOC					
≤2	27 (50)	52 (96.3)	0.07	0.02–0.23	<0.001
>2	27 (50)	2 (3.7)			
Number of blood transfusion					
≤1	45 (83.3)	53 (98.1)	0.11	0.02–0.85	0.016
>1	9 (17.7)	1 (1.9)			
Number of hospitalizations					
≤2	30 (55.5)	52 (96.3)	0.08	0.02–0.34	<0.001
>2	24 (44.5)	2 (3.7)			
Duration of hospital stay (days)					
≤7	17 (31.5)	51 (94.4)	0.08	0.02–0.24	<0.0001
>7	37 (68.5)	3 (5.6)			
Acute chest syndrome					
No	48 (88.9)	54 (100.0)	0.10	0.02–0.41	<0.001
Yes	6 (11.1)	0 (0.0)			
Haematologic					
	(Median/IQR)	(Median/IQR)	Z^a^		
Haematocrit (%)	26.0 (22.0–27.0)	28.0 (26.0–30.8)	−5.01	–	<0.0001
White blood cell count (×10^9^/l)	11.1 (7.3–14.6)	6.2 (4.7–10.4)	−5.08	–	<0.0001
Platelet count (×10^9^/l)	411 (272–492)	337 (246.5–400.5)	−2.78	–	0.005
Foetal Hb (%)	7.8 (4.6–12.9)	14.0 (11.7–20.3)	−6.39	–	<0.0001

a Wilcoxon signed ranks test.

HUT, hydroxyurea therapy; VOC, vaso-occlusive crisis; RR, relative risk; IQR, interquartile range; Hb, haemoglobin.

The number of participants who had more than two episodes of painful crises requiring parenteral analgesia reduced from 27 (50%) to 2 (2.7%; RR = 0.07; 95% CI = 0.02–0.23; *p* < 0.001). The risk of being transfused more than once was 0.11 times the risk of transfusion in the 12 months period preceding the hydroxyurea treatment (RR = 0.11; 95% CI = 0.02–0.85; *p* = 0.016). Similarly, the risk of hospital stay >7 days was 0.08 times the risk prior to the commencement of hydroxyurea (RR = 0.08; 95% CI = 0.02–0.24; *p* < 0.0001).

When the haematological parameters at baseline and 12 months after the commencement of hydroxyurea were compared, the median haematocrit level increased from 26 to 28% (*p* < 0.0001). The median percentage foetal haemoglobin level also increased from 7.8 to 14% (*p* < 0.0001), whereas the median white blood cell and platelet counts fell from 11.1 to 6.2 × 10^9^/l and 411 to 337 × 10^9^/l, respectively (*p* < 0.0001 and *p* = 0.005; Table 3).

### Clinical and haematologic events in the study population following hydroxyurea therapy

The clinical and haematological events recorded in the study population are as shown in Table 4. About a quarter of the subjects self-reported increased appetite and activity, while abdominal pain and diarrhoea were reported by three (5.6%) and two patients (3.7%), respectively. One episode each of passage of red urine which cleared spontaneously after 1 week (the event was not further investigated as it cleared before reporting) and hyperpigmentation of the skin, palms and nails were documented. None of the subjects had severe anaemia, while six (11.1%) and two of the subjects (3.7%) had leucopenia and thrombocytopenia, respectively. The dosage at which the patients developed leucopenia/thrombocytopenia ranged from 25 to 35 mg/kg (Table 5).

**Table 4:** Clinical and haematologic events in the study population following hydroxyurea therapy

Events^a^	Frequency (%)
Increased appetite	14 (25.9)
Increased energy	14 (25.9)
Abdominal pain	3 (5.6)
Diarrhoea	2 (3.7)
Vomiting	1 (1.9)
Headache	1 (1.9)
Passage of red urine	1 (1.9)
Hyperpigmentation of the skin, palms and nails	1 (1.9)
Generalized body aches	1 (1.9)
Leucopenia	6 (11.1)
Thrombocytopenia	2 (3.7)
Severe anaemia	0 (0)

a Multiple events were recorded.

**Table 5:** Summary of data of the subjects who had leucopenia and thrombocytopenia

Parameter/subject number	Age (years)	Sex (M/F)	Baseline cell count (×10^9^/l)	Reduced cell count (×10^9^/l)	HU dosage at occurrence (mg/kg)
Leucopenia					
1	4.5	M	5.6	3.0	25
2	11.0	M	5.4	3.3	30
	5.2	F	8.6	3.3	25
	12.8	F	9.5	2.9	30
5	8.0	M	8.0	2.6	35
6	8.0	F	7.0	3.0	35
Thrombocytopenia					
1	4.0	M	180.0	78.0	35
2	7.0	F	192.0	79.0	35

HU, hydroxyurea; M, male; F, female.

## DISCUSSION

The outcome of this study showed that hydroxyurea is effective in reducing the occurrence of vaso-occlusive crises, acute chest syndrome, blood transfusions, hospitalization and duration of hospitalization among the study participants. There was 46.3% reduction in the occurrence of painful crises in the subjects following hydroxyurea therapy. The rate of reduction of painful crises in this study was similar to what had been reported in several studies mostly conducted in developed countries [12, 16, 18]. A painful crisis is a significant morbidity among SCA sufferers and any intervention that can significantly reduce the severity and frequency of this problem is worthwhile. In this study, the risk of being hospitalized in the 12 months following initiation of hydroxyurea was significantly reduced compared with baseline. Similarly, when hospitalized, the duration of hospitalization was shorter when on hydroxyurea therapy compared with the baseline condition. The reduction in the frequency and duration of hospitalization can impact positively on the quality of life of these patients. It can also reduce the huge financial burden that is associated with the treatment of this condition especially in our setting where most of the sufferers pay out of their pockets as they are mostly not covered under the national health insurance scheme. The reductions in the frequency and duration of hospitalizations following hydroxyurea therapy had been previously reported by several studies [12, 16, 29]. In our study, the risk of being transfused and having episodes of acute chest syndrome also reduced significantly after hydroxyurea therapy compared with pre-hydroxyurea period. As it was found in this study, hydroxyurea has been documented to increase the haematocrit level in SCA patients thereby raising the threshold at which transfusion will be considered in these patients. Previous studies have also documented the reduction of the frequency of transfusions and acute chest syndromes among the populations studied [12, 18, 24, 29].

This study also demonstrated that leucocyte and platelet counts were reduced after hydroxyurea therapy compared with the baseline values. These findings agree with data from previous studies in adults and children [12, 29]. The reduction in the white blood cells and platelet counts is a good development. Elevated steady state leucocyte and platelet counts have been documented to be recognized triggers for the vaso-occlusive events occurring in SCA [30]. Lowering the circulatory levels of these cells, which are inflammatory biomarkers in SCD [31], reduces vaso-occlusive events in these patients. However, while relatively low cell counts are desirable, too low leucocyte and platelet counts could predispose the patients to the risk for infections and bleeding, respectively. Therefore, efforts must be made to ensure that the patients on hydroxyurea should have their cell counts monitored in order to detect these complications early. The median percentage foetal haemoglobin level was almost doubled in this study following the 12 months of therapy. The induction of foetal haemoglobin production has been reported to be the main mechanism of action by which hydroxyurea modifies the pathogenesis of SCA [32]. Increased foetal haemoglobin reduces the sickling process in sickle cell patients and thereby reduces the complications that are associated with this condition. Foetal haemoglobin level has also been reported to correlate with amelioration of the disease severity, such that high foetal haemoglobin is associated with low disease severity and vice versa [33].

This study has also demonstrated that hydroxyurea was safe among the studied population. A number of the patients experienced an increased level of energy and improved appetite which are thought to be related to the increased haematocrit level induced by hydroxyurea. The increased haematocrit level could have enhanced the oxygen carrying capacity in the body thereby improving the energy level of the subjects. Hackney *et al*. [34] previously reported an increase in both anaerobic muscular performance and aerobic cardiovascular efficiency in SCA patients on hydroxyurea compared with those who were not on hydroxyurea. The study concluded that hydroxyurea produces an improvement in the physical capacity of patients with SCA [34]. Further studies could be conducted to fully understand the mechanism of increased energy/activity in SCA patients on hydroxyurea.

Only a few participants reported gastrointestinal symptoms comprising of abdominal pain, vomiting and diarrhoea; and the symptoms were transient. These gastrointestinal symptoms are recognized side effects of hydroxyurea and they were not severe enough in this study to warrant cessation of medication. One of the subjects each reported an episode of passage of red urine and hyperpigmentation of the skin, palms and the nails. The causality of the red urine could not be determined. Nonetheless, hydroxyurea has been documented to cause hyperpigmentation of the skin, palms and the nails [24].

The haematologic toxicities of hydroxyurea seen among our subjects were leucopenia and thrombocytopenia. In our study, none of the subjects had severe anaemia and this is similar to the findings in the BABY‐HUG study where only 1% of the study participants had severe anaemia [29]. Hydroxyurea is known to increase haematocrit level as it has been demonstrated in this study but it could also cause pancytopenia. The leucopenia and thrombocytopenia that occurred in this study were transient, dose-dependent, baseline values-related but reversible. Most of the patients who had leucopenia developed it at a dosage that was ≥30 mg/kg, while the two patients who had thrombocytopenia developed the condition at 35 mg/kg. After hydroxyurea was discontinued for a week and recommenced at a dosage that was 5 mg/kg lower than the toxic dosage, the cell counts normalized for all the patients. It was also interesting to note that none of the patients who developed leucopenia and thrombocytopenia exhibited any incidence of infection or bleeding episodes.

The two of our subjects who had leucopenia while taking hydroxyurea at 25 mg/kg had low baseline leucocyte counts. No other subject had leucopenia at the hydroxyurea dosage that was <25 mg/kg. These findings underscore the importance of individualizing the monitoring of patients on hydroxyurea. The findings also corroborate the clamour for a low fixed dosage of hydroxyurea especially in settings where monitoring of patients is beyond the economic reach of the patients. A well-planned randomized control trial may be required to authenticate these observations.

One limitation of this study is the short duration of follow-up to identify adverse events, as some adverse events may take a longer duration to develop. A long-term prospective study of adverse events of hydroxyurea in a low-middle income country like Nigeria is desirable. The findings from our study hopefully lend itself to form a template for further and multicentre phase IV clinical study.

## CONCLUSION

This study has demonstrated that hydroxyurea therapy is effective in reducing the frequency of painful vaso-occlusive crises, acute chest syndrome, blood transfusions, hospitalizations and the duration of hospitalization among Nigerian children with SCA. It has also shown that hydroxyurea is safe among the study participants. A multicentre study is recommended to obtain more robust data on the effectiveness and safety of hydroxyurea across the different parts of Nigeria. The study outcome may help improve the level of utilization of hydroxyurea in the treatment of SCA children.

## AUTHORS CONTRIBUTIONS

This work was carried out in collaboration between all authors. A.O.D.O. contributed to the conceptualization, design, literature search, data collection and analysis, manuscript preparation, editing and review. S.O., T.O.A., A.I.Z. and A.S.S. contributed to the study design, data analysis, manuscript preparation, manuscript editing and manuscript review. K.A., E.S.O. and I.N.D. contributed to the study design, manuscript preparation, manuscript editing and manuscript review. All authors read and approved the final manuscript.
